# Ipsilateral Right Angular Pregnancy After a Laparoscopic Right Salpingo-Oophorectomy: A Case Report

**DOI:** 10.7759/cureus.46171

**Published:** 2023-09-29

**Authors:** Genichiro To, Keisuke Kodama, Ichiro Onoyama, Hideaki Yahata, Kiyoko Kato

**Affiliations:** 1 Department of Obstetrics and Gynecology, Graduate School of Medical Sciences, Kyushu University, Fukuoka, JPN

**Keywords:** intrauterine pregnancy, laparoscopic surgery in pregnancy, ectopic pregnancy, angular pregnancy, interstitial pregnancy

## Abstract

It can be difficult to distinguish an interstitial pregnancy from an angular pregnancy because of the close proximity of the implantation sites. The difference in pregnancy outcomes between interstitial and angular pregnancies makes this distinction very important. A 39-year-old gravida 7 para 4 who had undergone a laparoscopic right salpingo-oophorectomy (RSO) one year ago and a pregnancy termination via dilation and curettage (D&C) three weeks ago was suspected to have a ruptured right interstitial or angular pregnancy. The patient underwent a laparoscopic total hysterectomy. The postoperative histologic diagnosis was an abortion of a right angular pregnancy. Indeed, it is essential to rule out an interstitial or angular pregnancy during adnexal surgery, even soon after elective abortion. Proper management of an angular pregnancy could prevent a fatal outcome following a rupture or massive hemorrhage.

## Introduction

Ectopic pregnancies account for approximately 2% of all pregnancies [1,2]. Ectopic pregnancies that rupture can be fatal and have been reported to account for 2.7% of maternal deaths [3].

Pregnancies are classified as intrauterine or ectopic. Intrauterine pregnancies are classified into normal and angular pregnancies. An angular pregnancy is an eccentric intrauterine pregnancy with implantation of the embryo in the lateral superior angle of the uterine cavity [4-7]. An angular pregnancy is a rare condition, with < 100 cases reported in the literature [5].

Ectopic pregnancies are classified as tubal or non-tubal. Non-tubal pregnancies are further classified as ovarian (3%), interstitial (2.5%), abdominal (1.3%), cornual (0.27%), cervical (<1%), and cesarean scars (0.05%-0.4%) [8,9]. An interstitial pregnancy occurs when implantation is within the myometrial portion of the fallopian tube.

It can be difficult to distinguish an interstitial pregnancy from an angular pregnancy because of the close proximity of the implantation sites. The difference in pregnancy outcomes between interstitial and angular pregnancies makes this distinction very important. An angular pregnancy can progress to full term, although abortion, uterine rupture, and placenta accreta can occur. In contrast, interstitial pregnancies ultimately rupture or are terminated surgically or pharmacologically [4,10,11].

Herein, we report the case of a patient with an angular pregnancy in whom intraabdominal hemorrhage occurred without uterine rupture. The bleeding arose from a previous right salpingo-oophorectomy scar. There are some reports of interstitial pregnancies after ipsilateral salpingo-oophorectomy [12]; however, there are few reports of an angular pregnancy after an ipsilateral salpingo-oophorectomy.

## Case presentation

A 39-year-old female, gravida 7 para 4, had a positive pregnancy test on March 21, 2022. Her previous physician performed a pregnancy termination via dilation and curettage (D&C) on March 24. After the D&C, a levonorgestrel-releasing intrauterine system (LNG-IUS) was inserted for contraception. The patient presented to our department on April 15 for an evaluation of persistent and worsening upper abdominal pain.

Her surgical history included a laparoscopic right salpingo-oophorectomy (RSO) performed one year ago for a right ovarian mature cystic teratoma. The physical examination was significant for tenderness all over the lower abdomen. A vaginal speculum examination showed a small amount of brown discharge. The pelvic examination was significant for uterine motion tenderness. Transvaginal ultrasonography showed a cyst-like structure in the area of the right adnexa. The LNG-IUS was noted in the uterine cavity. The right adnexa had been surgically excised, and no abnormal findings were present in the left adnexa. There was a moderate amount of echo-free space in the pouch of Douglas.

**Figure 1:**
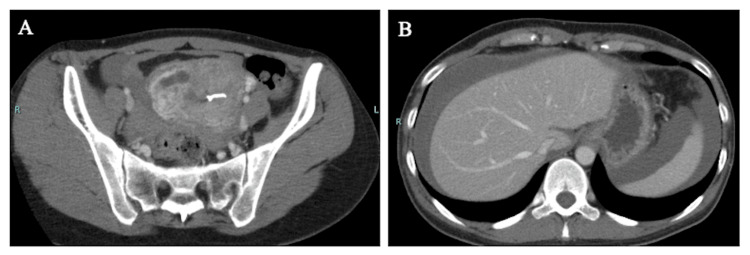
Contrast-enhanced CT of the upper abdominal and pelvic region (A) A 5 cm mass was noted on the right side of the uterus, and LNG-IUS was noted in the uterine lumen; (B) Ascites had accumulated up to the upper abdomen.

An upper abdominal-pelvic contrast-enhanced computed tomography showed a mass on the right aspect of the uterine body and an LNG-IUS in the uterine cavity. Ascites accumulated in the upper abdomen. No findings suggestive of appendicitis were noted (Figure 1).

The admitting diagnoses included suspected pelvic inflammatory disease, perforation of the uterus, and a moderate amount of ascites.

On the day of admission, she was treated with intravenous antibiotics, and the LNG-IUS was removed. On hospital day three, the severity of anemia had worsened (the hemoglobin decreased from 11.8 g/dL to 8.3 g/dL), so rupture of an ectopic pregnancy was suspected.

**Figure 2:**
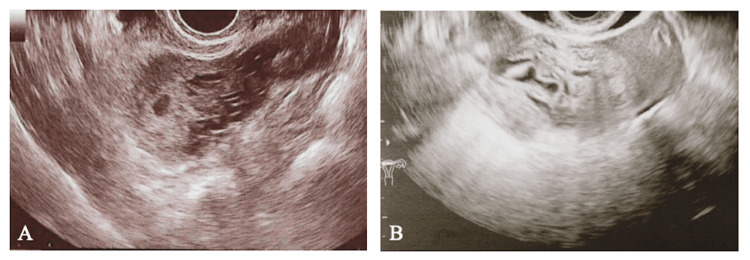
Transvaginal ultrasonography (A) Findings from the previous clinic: a hypoechoic 4 cm mass thought to be a gestational sac was observed in the right uterine horn; (B) Findings from our hospital: a heterogeneous 4 cm mass was observed in the right uterine horn.

Transvaginal ultrasonography (US) by the former physician showed a gestational sac (GS) in the uterus, but the US performed at our hospital was suspicious of a hematoma at the base of the uterus (Figure 2).

The GS had disappeared, but the possibility of persistent trophoblastic disease could not be ruled out because Doppler flow was observed at the same site. The serum human chorionic gonadotropin (hCG) was elevated to 24,073 mIU/ml.

**Figure 3:**
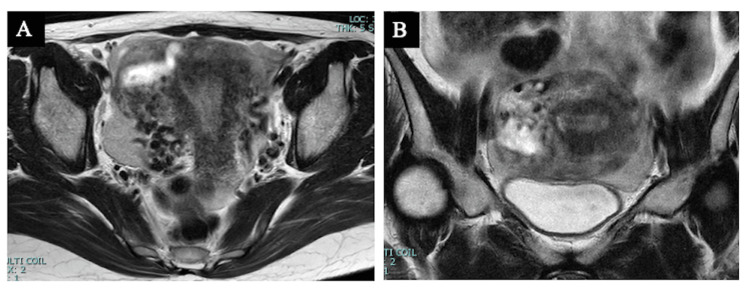
Pelvic contrast-enhanced magnetic resonance imaging (T-enhanced image) (A) Transverse plane; (B) Coronal plane; a mixed high-to-low signal intensity (5 cm) mass was found on the right side of the uterine body.

Pelvic contrast-enhanced magnetic resonance imaging (MRI) showed mixed high-to-low signals on T2-enhanced images and a 5-cm mass on the right side of the uterine body (Figure 3).

The MRI findings showed no staining from the early phase, which was suggestive of chorionic disease.

**Figure 4:**
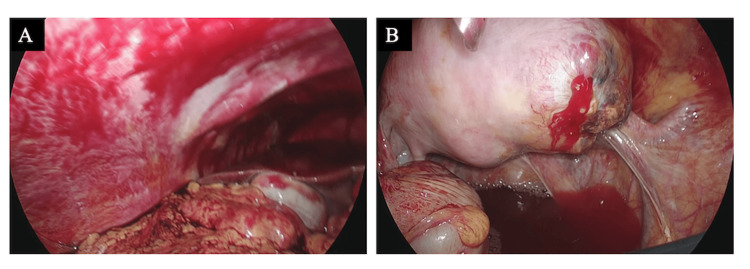
Laparoscopic findings (A) Bloody ascites accumulated on the subhepatic surface; (B) The origin of the right fallopian tube was swollen to the size of a thumb, and bleeding was observed.

Based on the above findings, the preoperative diagnosis was a suspected rupture of a right interstitial or angular pregnancy. The patient had no desire to preserve fertility, so we performed a laparoscopic total hysterectomy (conventional, diamond method, four-port). Intraoperatively, bloody ascites had accumulated on the subhepatic surface. The origin of the right fallopian tube was swollen to the size of the thumb, and bleeding was noted from the pelvic cavity (Figure 4).

**Figure 5:**
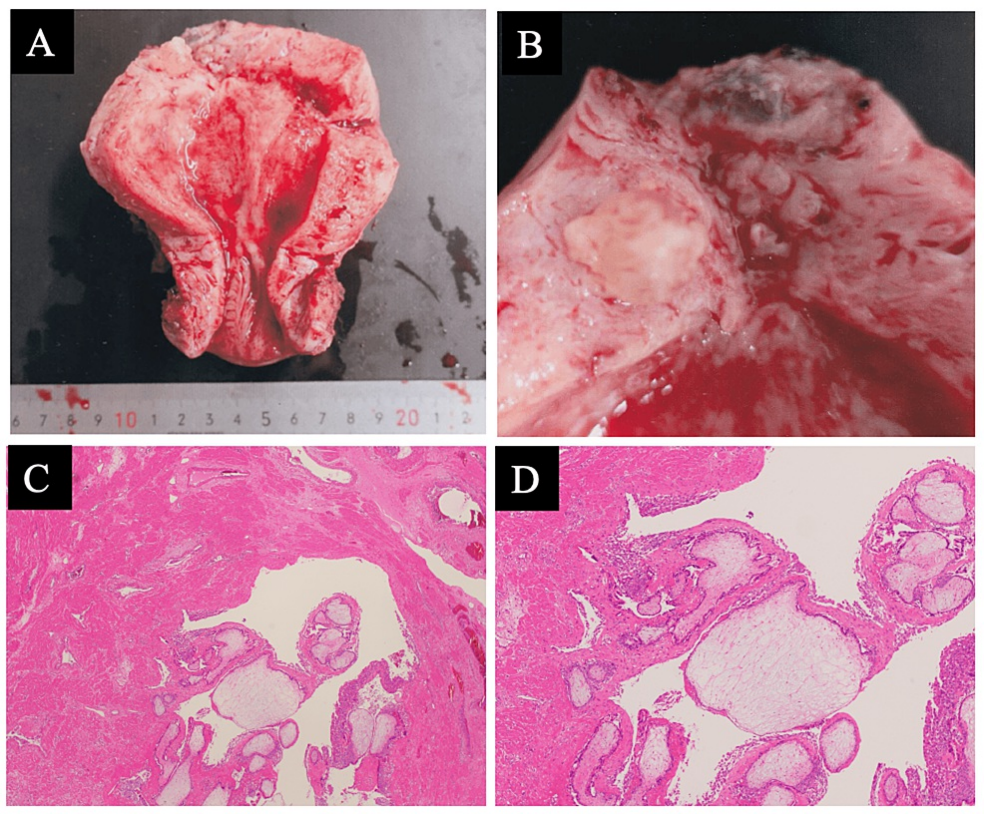
Macroscopic findings and pathologic findings (A) and (B) A dark brown 3 cm mass was found in the right fallopian tube interstitium; (C) and (D) Chorionic villi tissue was observed in the uterine myometrium; there was no tubal epithelium.

The surgical specimen showed a 3 cm-large mass in the myometrium of the right interstitial pregnancy. The pathologic examination revealed chorionic villi tissue within the uterine myometrium and no tubal epithelium (Figure 5).

The postoperative diagnosis was an abortion of an angular pregnancy. On the fourth postoperative day, the hemoglobin concentration was stable and serum hCG had decreased. On the tenth postoperative day, the patient was discharged home in stable condition.

## Discussion

We managed a case in which distinguishing between an angular and an interstitial pregnancy was difficult. A hysterectomy was performed because the patient had undesired fertility, but the treatment options would have differed if the patient had a desire to continue the pregnancy.

If the patient has a desire to continue the pregnancy, they should be carefully monitored. It has been reported that when following up, the diagnosis of an angular pregnancy should be reliably made using MRI in addition to ultrasound [13]. It is also important to explain to the patient the high risk of preterm delivery, premature abruption of the normal placenta, and FGR, as well as fatal uterine rupture, and to perform frequent ultrasound examinations. In some cases, inpatient management is also required [14,15].

An angular pregnancy implant is medial to the uterotubal junction, and it is possible to continue the pregnancy with careful observation [5,13,16]. In contrast, an interstitial pregnancy is implanted in the interstitial portion of the fallopian tube that lies in the uterine myometrium and has a high rate of maternal morbidity.

Although we were unable to identify the exact site of the pregnancy, we suspected a ruptured ectopic pregnancy because of the rapid worsening of anemia, the absence of a fetal sac in the uterus, a mass in the right adnexal region on transvaginal ultrasonography and MRI, and the high hCG level [17,18].

As a method to distinguish angular and interstitial pregnancies preoperatively, Monteagudo et al. proposed three ultrasound criteria to diagnose interstitial pregnancies, as follows: (1) an empty uterine cavity; (2) a chorionic sac separate (> 1 cm) from the lateral edge of the uterine cavity; and (3) a thin myometrial layer (< 5 mm) surrounding the chorionic sac. In this case, a GS was observed in the uterine cavity, and the chorionic sac was near the lateral edge of the uterine cavity, surrounded by a thick myometrial layer (> 5 mm) [19]. Furthermore, Jansen et al. [16] suggested the following clinicosurgical criteria to define an angular pregnancy: (1) clinical presentation with painful asymmetric enlargement of the uterus; (2) direct observation (i.e., surgical) of lateral uterine distention with lateral displacement of the round ligament; and (3) retention of the placenta in the uterine angle. In our case, asymmetric enlargement of the uterus and displacement of the round ligament laterally are observed. The placenta had not yet formed. Ultimately, the diagnosis can only be made histologically; however, there are many ways to make a tentative diagnosis preoperatively [6,15,16].

The patient had a right angular pregnancy after a laparoscopic right salpingo-oophorectomy. Initially, whether the pregnancy was angular or interstitial should be determined. If it is an interstitial pregnancy, the pregnancy should be terminated. If it is an angular pregnancy, the pregnancy can be continued if there is no severe abdominal pain or worsening anemia suggestive of incipient rupture. In our case, intra-abdominal bleeding was not due to a rupture of the myometrium but from the right salpino-oophorectomy wound. It is difficult to continue an angular pregnancy after an ipsilateral salpingo-oophorectomy because of the high risk of intra-abdominal bleeding [1,3,6,11,13].

## Conclusions

It is essential to rule out an interstitial or angular pregnancy during adnexal surgery, even after an elective abortion. It is important to differentiate between an interstitial and an angular pregnancy because an angular pregnancy can continue to term. However, an angular pregnancy after a salpingo-oophorectomy would make the continuation of the pregnancy difficult because a fatal outcome could occur, such as a rupture or massive hemorrhage.
